# Bevacizumab in recurrent high-grade glioma: a single institution retrospective analysis on 92 patients

**DOI:** 10.1007/s11547-021-01381-5

**Published:** 2021-06-03

**Authors:** Beatrice Detti, Silvia Scoccianti, Maria Ausilia Teriaca, Virginia Maragna, Victoria Lorenzetti, Sara Lucidi, Chiara Bellini, Daniela Greto, Isacco Desideri, Lorenzo Livi

**Affiliations:** Radiation Oncology Unit, Azienda Ospedaliero-Universitaria Careggi, Largo Brambilla 1, 50134 Florence, Italy

**Keywords:** High-grade glioma, Bevacizumab, Glioblastoma, Central nervous system

## Abstract

**Background:**

High-grade gliomas are among the most aggressive central nervous system primary tumors, with a high risk of recurrence and a poor prognosis. Re-operation, re-irradiation, chemotherapy are options in this setting. No-best therapy has been established. Bevacizumab was approved on the basis of two Phase 2 trials that evaluated its efficacy in patients with recurrent glioblastoma.

**Materials and methods:**

We have retrospectively review data of patients with high-grade glioma treated at our institution that undergone radiological or histological progression after at least one systemic treatment for recurrent disease. Bevacizumab was administered alone or in combination with chemotherapy until disease progression or unacceptable toxicity. Bevacizumab regimen was analyzed to assess PFS and OS. Histological, molecular and clinical features of the entire cohort were collected.

**Results:**

We reviewed data from 92 patients, treated from April 2009 to November 2019, with histologically confirmed diagnosis of high-grade gliomas and recurrent disease. A PFS of 55.2%, 22.9% and 9.6% was observed at 6, 12 and 24 months, respectively. Performance status, age at diagnosis (< 65 or > 65 ys.) and use of corticosteroids during bevacizumab therapy were strongly associated with PFS. The OS was 74.9% at 6 months, 31.7% at 12 months, 10.1% at 24 months. In our cohort, 51.1% were long-term responders (PFS > 6 months). Globally, bevacizumab treatment was well tolerated.

**Conclusion:**

Our analysis confirms the efficacy of bevacizumab in recurrent high-grade glioma patients with an acceptable toxicity profile, in keeping with its known safety in the literature.

## Introduction

High-grade gliomas are among the most aggressive primary tumors in central nervous system (CNS) and are correlated with poor prognosis despite treatments [1, 2]. Multimodal approach with surgery, radiation and/or chemotherapy is the standard of care. However, high-grade gliomas relapse in most cases, and a change of therapeutic approach is needed. Re-operation, re-irradiation, chemotherapy, alone or in combination, are options in this setting, although the best therapy has not been established and prognosis remains poor. Chemotherapy regimens frequently used are rechallenge with temozolomide, nitrosoureas (i.e., lomustine, carmustine or fotemustine) [3–6] and bevacizumab as single agents or in combination [7–10]. The overexpression of vascular endothelial growth factor (VEGF-A), along with microvascular proliferation and damage to the blood–brain barrier, is observed in patients with recurrent malignant gliomas. [11, 12] Bevacizumab (Avastin ®) is an IgG1 humanized monoclonal antibody against VEGF-A, used in recurrent clinical setting since 2009 [13]. Bevacizumab was approved on the basis of two Phase 2 trials that evaluated his efficacy in monotherapy or in combination with irinotecan in patients with recurrent glioblastoma (GBM). According to a Food and Drug Administration (FDA) analysis, the duration of tumor response in monotherapy was of 4.2 20 months in study AVF3708g and 3.9 months in study NCI 06-C-0064E [14, 15]. After that, several phase II and III studies investigated the use of bevacizumab alone or in combination with chemotherapy in patients with recurrent high-grade gliomas, showing an improved survival, despite a risk of serious side effects [11]. In this mono-institutional retrospective study, we evaluated efficacy and tolerability of bevacizumab in patients with recurrent high-grade glioma.

## Materials and methods

Patients were retrospectively selected from our institution’s database. Eligibility criteria for the study were age at diagnosis > or = 18 years old; histologically confirmed high-grade glioma (including glioblastoma, oligodendroglioma, anaplastic astrocytoma and oligoastrocytoma), firstly managed with radiotherapy or radio-chemotherapy; radiological or histological progression after at least one line of systemic treatment for recurrent disease; bevacizumab administration of 15 mg/kg every three weeks (10 mg/kg every two weeks in patients with poor performance status) alone or in combination with chemotherapy (irinotecan, fotemustine, lomustine) until disease progression or unacceptable toxicity, according to standard practice. Clinical assessment, including a neurologic examination, and blood test (hematologic, renal and hepatic functions) before chemotherapy administration were considered. Response to treatment was evaluated by gadolinium-brain magnetic resonance imaging (MRI) according to response assessment in neuro-oncology high-grade glioma criteria (RANO-HGG) [16]. Radiological evaluation was performed every 3 months or when clinically indicated. All adverse events were graduated according to Common Terminology Criteria for Adverse Events (CTCAE), version 4.0 [17].

### Statistical analysis

Median overall survival (OS) and median progression-free survival (PFS) was calculated by Kaplan–Meier method. PFS was defined as the period from beginning of bevacizumab until progression or death from any cause or to the last day of follow-up. OS was calculated from the date of first bevacizumab to the date of the most recent follow-up or death from any cause or the last day of follow-up. The Cox regression model hazards were used to assess the effect of factors identified as significant on survival analysis by Kaplan–Meier. Karnofsky performance status (KPS) at beginning of treatment, age, sex, previous re-irradiation/re-surgery, steroids therapy and diameter of disease was evaluated for OS and PFS by univariate analysis and multivariate analysis. In a subgroup analysis, we defined as long-term responders the patients with time on bevacizumab more than 6 months on the basis of data by FDA [14, 15].

## Results

We collected data from 92 patients, with histologically confirmed diagnosis of high-grade gliomas and recurrent disease, treated at the Radiation Oncology Unit of the AOU Careggi, Florence Italy, between April 2009 and November 2019. Patients characteristics are described in Table 1.

**Table 1:** Main 92 patients characteristics

Feature	Patients	%
*Sex*		
M	57	62
F	35	38
*Age at diagnosis*		
≥ 65 y	20	21.7
< 65 y	72	78.3
*Histology*		
Glioblastoma	71	77.2
Other (*)	21	22.8
*MGMT status*		
MGMT methylated	38	41.3
MGMT not methylated	34	36.9
Unknown	20	21.7
*IDH status*		
Mutation	6	6.5
Wild Type	9	9.8
Unknown	77	83.7
*1p19q codeletion*		
Codeletion	2	2.2
No codeletion	9	9.8
Unknown	81	88
*Primitive treatment*		
Postoperative radiotherapy	87	93.55
Concurrent TMZ	81	93
Adjuvant TMZ	72	
number cycle of Adjuvant TMZ	Median 6	Range 1–26

*Other includes histologies of oligodendroglioma, oligoastrocytoma, anaplastic astrocytoma

## Patients received partial resection, 41 patients radical exeresis, while a stereotactic biopsy alone was performed in nine patients.

Bevacizumab in recurrent setting was administered as shown in Table 2. At the beginning of bevacizumab, a KPS of 90–100, 70–80 and < 70 was highlighted in 30.4%, 51.1% and 18.5% of patients, respectively. Median follow-up was 8.3 months, (range 1.4–59.8). The median number of bevacizumab cycles was 8 (range 1–75). The mean treatment time was 7.5 months. At the time of analysis, 4.3% patients were still on bevacizumab therapy. During bevacizumab treatment, the best response according to RANO-HGG criteria was the following: stable disease in 22.5%, partial response in 34% and complete response in 2.1%. Bevacizumab achieved reduction of 25.7% of dexamethasone needs during treatment (basal median dose was 4 mg ranging from 1 to 16 mg); in addition, a noteworthy benefit in KPS during anti-VEGF therapy was noted in approximately 19.1% of patients.

**Table 2:** Bevacizumab features

Feature	Patients	%
*Line of systemic treatment*		
First	46	50
Second	30	32.6
More than second line	16	17.4
*Bevacizumab combination therapy*		
Monotherapy	67	72.8
Combination chemotherapy	25	27.2
Fotemustine	18	72
Lomustine	5	20
Irinotecan	2	8

A PFS of 55.2%, 22.9% and 9.6% was observed at 6, 12 and 24 months, respectively. At KM analysis, performance status, age at diagnosis (< 65 or > 65 ys) and use of corticosteroids during bevacizumab therapy were strongly associated with PFS. These factors significantly impacted on PFS at univariate analysis, whereas only the PS had a significant impact at multivariate analysis.

The OS at 6 months was 74.9%, at 12 months was 31.7%; at 24 months was 10.1%. At the KM analysis resulted in a statistically significant difference in OS for sex, performance status, use of dexamethasone and max diameter of disease.

The results of univariate analysis confirmed the significant effects of the above four parameters. Performance status and no use of dexamethasone significantly impacted on overall survival at multivariate analysis.

When bevacizumab was given as first, second or third line or after, the median OS was 9.6 months (95% CI 7.6–11.3), 8.9 months (95% CI 7.7–16.4) and 7.8 months (95% CI 6.8–13.4), respectively (*p* = 0.54). The median PFS was 6.9 months (95% CI 5.4–8.7) in first line, 6.3 months (95% CI 4.9–11.6) in second line and 6.6 months (95% CI 2.9–8.9) in third line and after (*p* = 0.78).

No significant survival difference was found between use of bevacizumab alone and in combinations with other chemotherapy agents: Median OS was 9.4 months (7.7–13.4) and 8.9 months (95% CI 7.2–11.7), respectively; median PFS was 6.9 months (95% CI 5.4–7.5) and 6.3 months (95% CI 4.7–8.6), respectively.

The grade III glioma group (*n* = 21) had median OS of 11.7 months (95% CI 7.5–17.3), versus median OS of 9 months (95% CI 8–9.9; *p* = 0.27) in GBM group (*n* = 71). Median PFS was 5.8 months (95% CI 2.9–13) in grade III glioma group and 6.9 months (95% CI 5.4–7.5) in GBM group (*p* = 0.94).

**Fig. 1:**
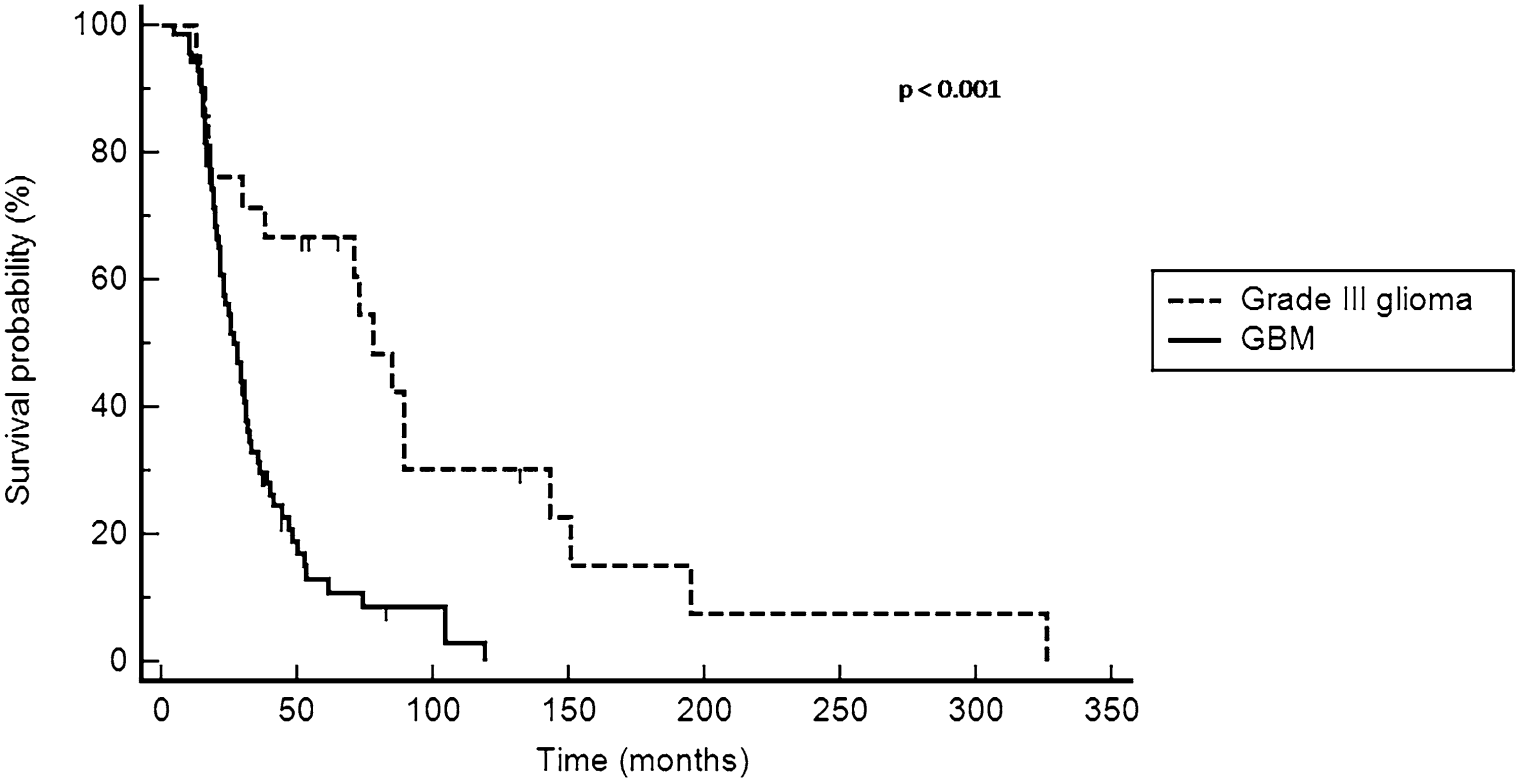
Overall survival from initial diagnosis in Grade III glioma group (*n* = 21) and glioblastoma (GBM) group (*n* = 71)

In a further sub-analysis, patients with grade III glioma showed better overall survival from initial diagnosis than these with GBM: The 3-year OS rate was 71% vs. 30%, respectively; the 5-year OS rate was 67% vs. 19%, respectively (*p* < 0.001; Fig. 1).

In GBM group (*n* = 71) the 6-month, 1-year and 2-year OS rate was 76.7%, 20% and 10%, respectively. The PFS rate was 58% at 6 months, 20% at 1 year and 11% at 2 years. No significant difference in survival was found for lines of bevacizumab treatment, MGMT methylation status or use of bevacizumab alone or in combined therapy regimen.

In our entire cohort, 51% (*n* = 47) were long-term responders (PFS > 6 months) and had GBM histology. Of these patients, 61% received bevacizumab monotherapy in first line, 34.1% in second line and 4.9% in subsequent lines. The median PFS was 38 weeks (26.9–265.7).

Only 6.5% of all population discontinued bevacizumab due to adverse effects. All of them had a thrombocytopenia, 50% G2 and 50% G3. In G2 cases, treatment discontinuation was decided because of concomitant hemorrhage G2 in one case, concomitant neutropenia G2 in one case and in another patient because of concomitant fatigue G2 and bowel perforation.

Concerning most known bevacizumab-related toxicities, G1 hemorrhage events occur in 4.3% of patients and only in 1 patient occurred hemorrhage G2, none of this regarding CNS district; hypertension G2 was detected in 3.3% of patients, one patient had a G1 and another one G3 hypertension. Only one case of intestinal perforation which led to a discontinuation treatment was recognized. Only two cases of G2 thrombosis occurred. Fatigue was a common event: 14.1% of the entire cohort manifested it. All reported toxicities occurred in the group of patients treated with the three-weekly regimen. Globally, bevacizumab treatment was fine tolerated, regardless of treatment lines and histological grade of tumor (see Table 3).

**Table 3:** Principal side effects occur during therapy with bevacizumab in all patients of study cohort, in patients of glioblastoma groups and in patients of glioma grade III groups

	All population group (*n* = 92)		GBM group (*n* = 71)		WHO III group (*n* = 21)		*p*	Odds ratio
	*n*	%	*N*	%	*n*	%		
Discontinuation	18	19.57	17	23.94	1	5.56	0.05	6.30
Thrombocytopenia	13	14.13	12	16.90	1	5.56	0.16	4.07
Anemia	0	–	0	–	0	–	–	–
Neutropenia	3	3.3	3	4.23	0	–	–	–
Lymphopenia	0	–	0	–	0	–	–	–
Nausea	2	2.17	1	1.4	1	–	0.35	0.29
Vomiting	0	–	0	–	0	–	–	–
High liver enzymes	2	2.17	2	2.81	0	–	–	–
Renal	3	3.3	2	2.81	1	5.56	0.66	0.58
Pulmonary	0	–	0	–	0	–	–	–
Cutaneous	1	1.09	1	1.4	0	–	–	–
Fatigue	13	14.13	11	15.49	1	5.56	0.20	3.67
Thrombosis	3	3.3	3	4.23	0	–	–	–
Hemorrhage	5	5.43	5	7.04	0	–	–	–

## Discussion

Our single-center retrospective experience of 92 patients with high-grade glioma receiving bevacizumab for recurrent disease revealed a median PFS of 26.9 weeks (5–265.7) and a median OS of 49.9 weeks (8.1–256.4). Restricting analysis on GBM histology subpopulation median PFS was 26.9 weeks (5.6–265.7), and median OS was 38 weeks (8.3–256.4). The majority of patients in our cohort, 67 (72.8%) received monotherapy, and only 25 (27.2%) received polychemotherapy, mostly with fotemustine. The literature review demonstrates a median PFS with monotherapy bevacizumab administration in recurrent setting ranging from 13 to 18.3 weeks and a median OS ranging from 31 to 40 weeks [12]; when bevacizumab is used in combination with a secondary agent, data attests around a median PFS of 8 to 25.6 weeks and a median OS of 15–52.1 weeks [18–21]. Data found in our experience accord to known survival rates with use of bevacizumab in recurrent high glioma.

We classified 47 cases as long-term responders, defined as patients with more than 6 months on bevacizumab treatment; not surprising, all of them were young patients (media 51.6 years), more than half had a complete surgical resection at first treatment and all were GMB histotype, with 48.9% mMGMT assessed at diagnosis. 57.4% of this population had a KPS more than 80 at the beginning of treatment, and 93.6% of them maintained the same quality of life during and at the end of treatment. In addition, most of them (61%) received bevacizumab as first-line at recurrence, 34.1% as second-line and only 4.9% in subsequent lines. In this subpopulation, we found a median PFS of 38 weeks (26.9–265.7) and a median OS of 57.4 weeks (29.3–256.4).

A multicentric retrospective study found a median PFS of 21.7 months and a median OS of 31.1 months in a subgroup of long-term responders with time on bevacizumab > 12 months [22]. These data support the role of bevacizumab in recurrent setting. Performance status was strongly associated with both PFS and OS. At multivariate analysis, performance status significantly impacts on PFS, while multivariate analysis for OS showed concomitant additional effect of sex, dexamethasone use and maximum diameter of primary lesion < 42 mm [23]. In our cohort of patients, we achieved a reduction of 25.7% of corticosteroids use, despite basal dexamethasone use before bevacizumab was low. This, again, could be related to performance status maintenance: Bevacizumab could act as corticosteroid-sparing agent decreasing peritumoral edema. Improvements in performance status consequent to anti-VEGF therapy and its relationships to survival outcomes have been already investigated in the literature [8, 24]. Similar to our experience, Annick Desjardins et al. observed a relationship through OS and also PFS and steroids dependance in 74 bevacizumab treated patients, in particular a median OS of 13.2 months in patients naïve from steroids and 7.2 in a subgroup who required supportive anti-edema therapy and corresponding PFS of 8.6 months and 3.7 months [25].

In our analysis, patients showed a 42.4% of methylation of MGMT gene; not surprising, the 56.4% of them were inscribed in long-term bevacizumab responders. The prognostic and predictive role of MGMT methylation is well known in high-grade gliomas treated with temozolomide, but hypermethylation seems to be also related to anti-VEGF treatment. A recent analysis published in NEMJ in 2017 by Wolfang et al. demonstrated doubling PFS time in MGMT methylated gliomas receiving bevacizumab in monotherapy, versus not methylated ones (2.8 vs. 5.7 months), [26].

Bevacizumab treatment was well tolerated, and the toxicity profile was safe despite systemic treatment line and combination with other anticancer drugs. Only the 6.5% of the entire population discontinued treatment because of toxicity. No unexpected events occurred. Fatigue and thrombocytopenia were the most frequent adverse effects found, and thrombo-hemorrhagic events were very low and never graded more than G2. The only G3 toxicity found was hypertension, rapidly medically managed without consequences for the patient. Of note, no CNS events were registered. The adverse effect profile was in line with previous experiences [14, 27].

Our study accounts for one of the bigger cohorts of recurrent GBM patients treated with bevacizumab; despite it is a monocentric retrospective study, our center is a high experience center that count on a multidisciplinary team of experts in CNS diseases, with weekly discussions; this work pictures a real-life experience, without legislative limitations. In fact, a legislative consensus of the single Tuscany region left us free to prescribe bevacizumab even if not AIFA (Agenzia Italiana del Farmaco) refunded. Limitations of our study are obviously the retrospective design, the heterogeneity of histological subtypes, with a 20% of not glioblastoma histotype, the fact that bevacizumab was used in different timings during the natural history of high-grade gliomas and polychemotherapies. Furthermore, in HGG patients treated with anti-VEGF such as bevacizumab, a pseudo-response may be observed on imaging. This is defined as a reduction in contrast enhancement without a true radiological response, leading to high response rates on PFS, but with no influence on OS [28]. To date, no validated predictive tumor markers identify a subset of patients that might benefit from this drug. A predictor of good outcome and response could be detected by analyzing gene expression profiles of glioblastoma patient tumors with a durable response [29, 30]. In recurrent malignant gliomas, the up-regulation of the VEGF pathway led to development of new target anti-angiogenic therapy regorafenib [31].

## Conclusion

Our results support safe bevacizumab use in recurrent high-grade glioma with impact on survival endpoints and acceptable toxicity profile. These data require further confirmation from prospective studies to incorporate new biomarkers that might help us to better select patients.
